# Prevalence of male partners involvement in antenatal care visits – in Kyela district, Mbeya

**DOI:** 10.1186/s12884-019-2475-4

**Published:** 2019-09-02

**Authors:** Elizabeth Kabanga, Alfred Chibwae, Namanya Basinda, Domenica Morona

**Affiliations:** 1 Catholic University of Health and Allied Sciences-Bugando, P.O Box 1464, Mwanza, Tanzania; 2 School of public health, Catholic University of Health and Allied Sciences P. O BOX 1464, Mwanza, Tanzania

**Keywords:** Antenatal care, Maternal and child health, Male partner involvement

## Abstract

**Background:**

In most countries in the world, promotion of maternal and child health is perceived as women’s role and men do not feel that they are responsible and see no reason to accompany their partners to Antenatal Care (ANC) clinics [Vermeulen, E., et al., BMC Pregnancy Childbirth 16:66, 2016]. Male involvement in Reproductive, Maternal, Neonates and Child and Adolescent Health (RMNCAH) programs in Tanzania is low. In Prevention of Mother to Child Transmission (PMTCT) program, the data shows only 30% attend couple counseling and only 8% for HIV counseling with their partners. There is limited data on prevalence of male involvement in ANC visits in Kyela. The purpose of this study was to determine prevalence of male involvement in ANC services and assess factors influencing male partners’ involvement in ANC visits in Kyela district in Mbeya. The findings from this study will serve as a baseline in efforts to increase male involvement in ANC care in Kyela.

**Methods:**

Hospital based cross-sectional study was undertaken in Kyela district, Mbeya from October 2017 to November 2017. Data was collected using structured questionnaire and analyzed using SPSS version 20. Factors with *P* values of < 0.05 in univariate logistic regression were included in a multivariable logistic regression model to determine predictor variables that are independently associated with the outcome. Significant difference was defined as a P- value less than 0.05 and Odds Ratio (OR) that did not include 1.0.

**Results:**

About 174 pregnant women who were visiting the ANC in their second to fourth visits or higher. About, 56.9% (99) attended with their male partners and 51% (52) of these reported to be accompanied by male partners to ANC because the women had requested their partners to accompany them. Attendance of male partners to ANC was significantly associated with male partner awareness of ANC visiting dates OR 24.1, 95% CI 6.8, 86.5, and *P* < 0.0001.

**Conclusion:**

Prevalence of male attendance to the ANC in Kyela district is not adequate as fearing of HIV testing seemed to decrease male attendance to ANC services. So, there is high need to improve ANC health services with a focus on male friendly services.

**Electronic supplementary material:**

The online version of this article (10.1186/s12884-019-2475-4) contains supplementary material, which is available to authorized users.

## Background

Worldwide, male presence in Antenatal care (ANC) and during delivery remains a challenge to safe motherhood [1]. Each year 210 million women become pregnant, 15% of these develop complications and over half a million die due to reasons related to pregnancy or birth [2]. Statistics of World Health Organization (WHO) indicate that 99% of all maternal deaths occurs in low and middle-income countries; almost half of these occur in sub-Saharan Africa, [1, 2].

Promotion of maternal and child health is perceived as women’s role and men do not feel that they are responsible and see no reason to accompany their partners to ANC visits [3]. Male participation in antenatal care is thought to be an essential step leading to positive maternal and new born health outcomes [4]. Although there is active promotion of male involvement during antenatal care, a small number of men in Tanzania with their wives during antenatal care visits. In Sub-Saharan Africa, the non-attendance of male partners to ANC services is due to; health care staff treatments of male partners, prolonged waiting time during ANC visits and a loss of income if men accompany their partners in ANC [5, 6]. A fear of HIV/AIDS as reported in Rwanda as a barrier to scaling up the provision of antenatal care, men who recognize or believe that they are HIV positive are less willing to go together with their partners to antenatal clinics for testing [3, 6]. A study done on acceptability and preferences among men and women for male involvement in Antenatal Care revealed that socio-cultural factors are the main barrier to antenatal male partner HIV testing [5].

Regardless of men’s positive attitudes; lack of male-friendly health infrastructure and inadequate understanding, by both community members and health facility providers in relation to the role of men during pregnancy impede their attendance [4].

Male involvement in child-bearing decisions is important and also has a positive impact on the suitability of Prevention of Mother To Child Transmission of HIV (PMTCT) interventions [7, 8]. Appropriate medical information to men is very important with regards to PMTCT interventions. First, well-informed men will be more likely to participate positively in the decision making for the well-being of the couple [7]. Next, women in the company of supportive partners will be more motivated to undergo HIV testing, to return for the HIV test result and to reveal the HIV result to their partners. Third, well-informed couples may be more likely to accept a low risk behaviour and increase shared support, regardless of the test results [7, 8].

The Tanzanian Ministry of Health, as well as WHO emphasizes the particular value of male involvement in ANC [4]. According to Tanzania one plan II 2016 PMTCT program, the data shows only 30% of women come for couple counseling with their partners [9, 10]. And a study done in Sub-Sahara Africa revealed that, men in low and middle income countries are the main decision-makers, decide women’s access to maternal health services and thus influence their health outcomes [11]. It has been suggested that, maternal health interventions, aiming to emphasize health care seeking behavior during ANC and birth, should reconsider male partner involvement and increase their opportunity on attending with their partners for the ANC services [4]. Improving facility and health providers attitudes toward male involvement has the potential to get better service delivery to all pregnant women; on the other hand, care must be taken to avoid discriminating against unaccompanied women who are either single or in unstable relationships [5].

While the barriers and challenges to male involvement in ANC services has previously been studied, until now, there has been no study of male attendance in ANC in Kyela district of Tanzania. The aim of this study is determine the prevalence of male involvement in ANC and to understand why men do not accompany their partners during ANC visits in Kyela districts.

## Methods

### Study area and design

This study was conducted in Kyela district in Mbeya region. The district has for population of 221,490, among these, females were 115,478 and males were 106,012.Agriculture is the major economic activity in the district where 88% of the population is involved in food and cash crop production. The district has 29 government dispensaries, 11 private dispensaries, 1 private hospital and 1 Government Hospital located at the district headquarters which serves as the main referral point for the whole district [9]. In the year 2016, there were 23,268 women aged 15 to 45 years and maternal mortality rate was 76/100,000(District Health Information System DHIS2). Also in the year 2016, of the 8547 women who became pregnant, those who attended ANC services were 52% for the first visit, 30% for second or third visit and only 17% visited ANC clinics four times (District Health Information System DHIS2). A hospital-based cross-sectional study was conducted at Kyela district. One hundred six pregnant women were recruited from Njisi Health centre and the remaining 64 pregnant women were recruited from Kyela district hospital from October 2017 to November 2017.

### Data collection

Semi-structured questionnaire was used to collect data on social demographic characteristics including age, educational level, occupation, marital status, religion and parity. Also, data on factors that hinder men’s participation to the ANC services as well as services provided to the couple attending were collected Additional file 1.

### Data analysis procedure

Collected data was entered and analysed using SPSS version 20 Chicago, IL: SPSS Inc. version 20. Sociodemographic data were summarised in the form of proportions. Univariate logistic regression was carried out to determine factors associated with male involvement in ANC visits. Factors with *P* values of < 0.05 were included in a multivariable logistic regression model to determine predictor variables that were independently associated with the outcome. Significant difference was defined as P- value less than 0.05 and Odds Ratio (OR) that did not include 1.0.

### Ethical consideration

Ethical approval was sought from the joint CUHAS and Bugando Medical Center Research Ethics Committee with ethical clearance number CREC/250/2017. Permission was obtained from Kyela District Executive Director and health facility administration to implement the study. The respondents were adequately informed using the participant’s informed written consent statement about all the relevant aspects of the study, including its aim, interview procedures, anticipated benefits and potential hazards.

## Results

### Sociodemographic characteristics of included participants

About 174 pregnant women who were visiting the ANC in their second to fourth visits or higher with Mean age of 27.0 (sd ±6.4) years and mean number of 1.8 children (SD ±1.4) participated in the study. 61.5% (107/174) were coming from rural areas, 81.0% (141/174) were Christians, 60.3% (105/174) had primary education, and 62% (102/174) were small scale farmers (Table 1).

**Table 1:** Social demographic characteristics of included participants

Response	Frequency (n)	Percentage (%)
Residence		
Urban	67	38.5
Rural	107	61.5
Religion		
Christian	141	81.0
Islam	33	19.0
Education		
Non-educated	12	6.9
Primary	105	60.3
Secondary education	45	25.9
College education	12	6.9
Occupation		
Small scale farmers	112	62.1
Business	41	23.6
civil servant	21	12.0

### Male partners’ attendance to antenatal care clinics

According to women who completed questionnaire, 56.9% (99) of male partners attended to the ANC services with their partners. Though 99 men were reported to have accompanied their partners to ANC services, only 90.9% (90) were confirmed from the ANC cards. About 51% (52/99) of all partners accompanying pregnant women were invited by their female partners to go together to ANC clinics. Of the participant 40.2% (41/99) of attended male partners attended as it was a requirement of the government to attend. However, 73.0%(127/174) of the participant reported that their male partners were aware of the female partner schedule and the ANC visiting dates and 86.8%(151/174) provided financial support for the ANC visit. Most of the respondents 94.4% (165/174) reported to believe that, their partners heard about ANC services. Among of them, about 64.4% (105/174) heard from their partners.

### Reasons why men did not attend to the ANC services

In this study, the most common reasons why male partners did not attend with their partners in the ANC clinics included fearing of HIV testing (18.7%), polygamy (14.7%), long queues (12.2%), and their partners not living together (10.7%).

This study found a significant association between male attendance to the ANC and knowing the dates for the ANC visits (OR: 18.7 95% CI: 7.2 to 48.0 P = < 0.0001), couple communication (OR 4.295% CI: 1.6 to 10.6,*P* = 0.003), hearing about ANC (OR 11.7,95% CI: 1.4 to 95.7 *P* = 0.02), and knowing about ANC services (OR: 3.6 95% CI: 1.5 to 8.8 *P* = 0.06,**)** Table 2. However in multivariate analysis the only significant factor found was if the partner know ANC visiting dates (OR 24.1, 95% CI 6.8, 86.5, and *P* < 0.0001), Table 3.

**Table 2:** Univariate logistic regression of factors associated with male attending ANC services

Responses	OR (95% C.I.)	*P* value
Residence		
Urban	Reference	0.72
Rural	0.9 (0.5,1.7)	
Religion		
Christians	Reference	0.28
Moslems	1.5 (0.7,3.3)	
Education level		
Informal	Reference	
Primary	0.6 (0.2,2.1)	0.45
Secondary education	0.4 (0.1,1.3)	0.12
College or higher education	0.4 (0.1, 1.9)	0.22
Occupation		
Peasants	Reference	
Business	0.6 (0.3, 1.2)	0.14
Civil servant	0.2 (0.1, 0.7).	0.01
Partner know ANC visiting date		
Yes	Reference	< 0.0001
No	18.7 (7.2, 48.0)	
Couple Communication		
Yes	Reference	0.003
No	4.2 (1.6, 10.6)	
Partner know About ANC care services		
Yes	Reference	0.005
No	3.6 (1.5,8.8)	
Partner heard about ANC services		
Yes	Reference	0.02
No	11.7 (1.4, 95.7)

**Table 3:** Multivariable logistic regression of factors associated with male attendance to ANC services

	OR (95% C.I.)	*P* value	Overall *P* value
Occupation			
Peasants	Reference		0.27
Business	0.9 (0.4, 2.1)	0.79	
Civil servant	0.3 (0.1, 1.3).	0.11	
Partner know ANC visiting date			
Yes	Reference	< 0.0001	
No	24.1 (6.8, 86.5)		
Couple Communication			
Yes	Reference	0.13	
No	0.24 (−0.04, 1.5)		
Partner know About ANC care services			
Yes	Reference	0.45	
No	1.7 (0.4,6.9)		
Partner heard aboutANC services			
Yes	Reference	0.49	
No	2.4 (0.2, 28.6)		

## Discussion

### Prevalence of male attending ANC services with their partners

Male participation in antenatal care is thought to be important in improving positive maternal and new born health outcomes [4]. In this study, the prevalence of men attending ANC visits with their partners was found to be 56.9%. This prevalence is higher than those reported from other studies done in Mbeya, Tanzania [12]. The former study reported that, only 39% of the facility have been receiving male partners for both ANC/PMTCT and that reported by government of Tanzania in one plan II of 2016 which was only 30% [12]. This can be explained with vast of improvement in national reproductive policies, infrastructures as well different modalities of reproductive health promotions. For examples pregnant women would not receive ANC services if they would not attend with their partners and those who attended with their partners will get priority services.

In contrast to our study which was conducted in rural areas, most studies done in either urban setting or referral health facilities in Africa had low prevalence of male attendance to the ANC services. Study done in Mwanza city, north western Tanzania reported that only 24.7% of mothers had their male partners involved in PMTCT which is a crucial component of ANC services [13]. Another study done in northern Tanzania had reported that few men attended the ANC-VCT services during prenatal compare to those attended during post natal care (12.5% vs. 40%) which also is lower than the findings from this study [14]. A study by Byanmugisha et al. in Uganda reported that, 26% of men whose wives were attending ANC at Mbale Regional Referral hospital reported to have full male involvement in ANC services [15]. In most urban areas there is large number of people with occasional jobs so have less habit for seeking health services. Men who had occasional job were less likely to participate in ANC than peasants in Kenya [16].

However, apart from attending to the ANC services, majority of men provided financial support to their partners for the attendance to the ANC services. This can be through paying for transport, accompanying their partner to ANC, joining inside the rooms as well as participating in different ANC services [4]. Most men support women’s attendance to the ANC even though their own attendance at the services is low [4]. This may be due to cultural system in most of Sub-Saharan Africa, men are the main decision makers in the family including for economic affairs as well as health issues [17–21]. This system can be used to motivate men to attend to the ANC services with their partners.

### Services provided in ANC services

In this study, men who attended ANC services did not only accompany their partners but about 97% of those who attended reported that they availed of all the ANC services in the particular clinics, this included voluntary counselling and HIV testing for the prevention of maternal to child transmission of HIV/AIDS and STIs/STDs. From other studies, poor male participation of male in ANC services were due to poor access, stigma and confidentiality of services that were unfriendly to men [22]. As reported in studies done in Tanzania, there is minimal health education and counselling during ANC visits. This is due to either high number of the clients in the clinics and long-time of waiting. Also, some of the clinics reported to have male unfriendly facility environment and poor health workers attitudes towards men. These discourages male partner participation in ANC services [23, 24]. In Uganda reported that the number of the educated men attending to the ANC were twice as much as those who were less educated [15]. However, in other studies financial constraint such as cost of travel to ANC were reported as the setback to the male attendance to the ANC [25, 26].

This study has found the significant association between couple communication with male partner attendance to the ANC services. This findings is similar to the findings reported in other studies in aspect that women were likely to bring their partners to ANC services especially after bringing their feedback of the services they obtained during previous ANC visit [13, 25, 27]. Verbal communication seems to lighten the mind of the male partners on the advantages of attending to the ANC with their partners. As reported in study done by *Chibwae* et al in Shinyanga 2014, couple communication about ANC helps men to increase male partner awareness about ANC services [3]. And hence, they were able to know about what happened on previous ANC visit and next ANC visiting dates which were all significant associated with their attendance in this study.

### Reported factors hindering male partners attendance to the ANC by pregnant women

For the women whose partners did not attend, different reasons were given out but majority were due to the fearing HIV testing. This reason was also reported in the study done in Uganda that male partners were concerned with stigma and confidentiality of services that were unfriendly to men after knowing their status [22, 28, 29]. Other studies also done in Tanzania reported to fear of HIV testing as a barrier for the male partner attendance to ANC services [13, 30]. So there is a need to improve campaigns for HIV counselling and testing for men in Tanzania and other parts of the world.

Another reason reported by women whose husbands did not attend at ANC was polygamy or partners are not living together. Similar data findings were reported in other different studies that reduces number of male attendance in ANC services [31, 32].

Health facility factors also have being implicated in this study such as long queue and long waiting time. Long waiting time and long duration of ANC clinics were also common barrier for male attendance to the ANC services reported in other studies [4, 33].

## Conclusions

This study has found that more than a half of the pregnant women who attended to the health facility attended with their male partners. Women played a great role in influencing their partners to participate in the ANC services and they invited their partners and they knew about the ANC services and schedule. So women should be encouraged more to communicate with their male partners and encourage them to attend to the ANC. Couple communication and hearing about ANC services were significantly associated with male attendance to the ANC services.

## Additional file

Additional file 1: English version questionnaire. Ethical clearance latter (DOCX 16 kb)

## Data Availability

The datasets used and/or analyzed during the current study are available from the corresponding author on request.

## Abbreviations

ANC: Antenatal care
BMC: Bugando Medical Centre
CUHAS: Catholic University of Health and Allied Sciences-Bugando
DHSI2: District Health Information System
FP: Family Planning
PMTCT: Prevention of Mother to Child Transmission
VCT: Voluntary Counseling and Testing
WHO: World Health Organization
