# Supplementary material for: Prevalence of male partners involvement in antenatal care visits – in Kyela district, Mbeya
Source: BMC Pregnancy Childbirth. 2019 Sep 2;19:321. doi: 10.1186/s12884-019-2475-4 (PMC6720074; doi:10.1186/s12884-019-2475-4)
Supplement: Supplementary file 1 — English version questionnaire. Ethical clearance latter (DOCX 16 kb) [file 12884_2019_2475_MOESM1_ESM.docx]

# Appendix 1: English Questionnaire

Please provide information on the following questions by writing the coding number in respective section.

Questionnaire ID No:

Date of Interview: ________________________

Interviewer’s Name: ______________________

| **Questions** | **Coding** |
| --- | --- |
| **A: SOCIO-DEMOGRAPHIC INFORMATION** |  |
| 1. What is your Age (in Years) | [ ] |
| 1. What is your place of Residence? |  |
| 1. What is your religion? |  |
| 1. What is the highest level of education did you attain 2. = informal education, 2 = Primary Education 3 = Secondary Ed., 4 = higher education. | [ ] |
| 1. What is your current main occupation?   1 = small scale farmer; 2 = business; 3 = civil servant/public services; 4 = others specify | [ ] |
| 1. How many living children do you have? | [ ] |
| **B.MALE INVOLMENT** |  |
| 1. Does your partner attend antenatal care with you?   0 = Yes; 1 = No | [ ] |
| 1. How many times? | [ ] |
| 1. Does your partner know your antenatal appointment   0=Yes; 1=No  10. Does your partner discuss antenatal intervention with you?  0=Yes; 1=No | [ ] |
| 11. Does your partner support your antenatal visits financially?  0=Yes; 1;No | [ ] |
| 12.Does your partner take time to find out what goes on in antenatal care  0=Yes; 1=No | [ ] |
| **C**:**KNOWLEDGE ON ANTENATAL CARE SERVICES** | [ ] |
| 13.Have your partner ever heard about antenatal care services  0 =Yes; 1 = No | [ ] |
| 14. May you show me an antenatal card record?  1.confirmed 2.not confirmed | [ ] |
| 15.If attended, which service did you and your partner get?(mention) |  |
| a.HIV testing and counselling  0=Yes; 1=No | [ ] |
| b. Sexual transmitted disease testing  0 = Yes; 1 = No | [ ] |
| c.Family planning services  0 = Yes; 1 = No | [ ] |
| d.Birth preparedness  0 = Yes; 1 = No | [ ] |
| 16. Why did your partner attended to antenatal care service?  1.invited my partner  2.See other men attended with their partners  3.it is a law to attend with my partner to have a service at health facility | [ ] |
| **D**.**DETERMINANTS OF MALE PARTINERINVOLVENT INANTENATAL CARE CLINICS** | [ ] |
| 17.If your partner did not attend the antenatal care with you, what is the reason stopping you from attending ANC services | [ ] |
| - - - - 1. Antenatal care are just for woman only   0 = Yes; 1 = No | [ ] |
| - - - - 1. It is against faith/religion/tradition   0 = Yes; 1 = No | [ ] |
| - - - - 1. He don’t have enough information about antenatal care   0 = Yes; 1 = No | [ ] |
| - - - - 1. Relative and peers pressure   0 = Yes; 1 = No | [ ] |
| - - - - 1. Nurse and health workers are not welcoming to male partner   0 = Yes; 1 = No | [ ] |
| - - - - 1. Long queue waiting for health facilities   0= Yes; 1 = No | [ ] |
| g, He is a polygamy partner  0=Yes; 1=No | [ ] |
| h. we do not live together  0=Yes; 1=No | [ ] |
| I .I did not ask him to escort  0=Yes; 1=No | [ ] |
| j. partner busy  0=Yes; 1=No | [ ] |
| k. I do not want escort  0=Yes. 1=NO | [ ] |
| J.ANC clinic is too far  0= Yes; 1= No | [ ] |
| **E,INTENTION TO USE ANC IN FUTURE** |  |
| 18.Do you intend to recommend to your partner to visit ANC with you in the future  0=Yes; 1=No | [ ] |
| 19.Do your partner plants to visit ANC in the future  0=Yes, 1=No | [ ] |
| 20.do you wish for your partner to attend ANC with you  0=Yes; 1=No | [ ] |
| 21.why Yes/ why No |  |
